# Cardiomyocyte Progenitor Cells as a Functional Gene Delivery Vehicle for Long-Term Biological Pacing

**DOI:** 10.3390/molecules24010181

**Published:** 2019-01-05

**Authors:** Anna M. D. Végh, A. Dénise den Haan, Lucía Cócera Ortega, Arie O. Verkerk, Joost P. G. Sluijter, Diane Bakker, Shirley van Amersfoorth, Toon A. B. van Veen, Mischa Klerk, Jurgen Seppen, Jacques M. T. de Bakker, Vincent M. Christoffels, Dirk Geerts, Marie José T. H. Goumans, Hanno L. Tan, Gerard J. J. Boink

**Affiliations:** 1 Heart Center, Clinical & Experimental Cardiology, Amsterdam University Medical Centers, Academic Medical Center, 1105 AZ Amsterdam, The Netherlands; a.m.d.vegh@lumc.nl (A.M.D.V.); denisedenhaan@gmail.com (A.D.d.H.); l.coceraortega@amc.uva.nl (L.C.O.); a.o.verkerk@amc.uva.nl (A.O.V.); diane.bakker@amc.uva.nl (D.B.); s.c.vanamersfoorth@amc.uva.nl (S.v.A.); m.klerk@amc.uva.nl (M.K.); j.m.debakker@amc.uva.nl (J.M.T.d.B.); h.a.geerts@amc.uva.nl (D.G.); h.l.tan@amc.uva.nl (H.L.T.); 2 Cell and Chemical Biology, Leiden University Medical Center, 2333 ZA Leiden, The Netherlands; M.J.T.H.Goumans@lumc.nl; 3 Department of Cardiology, Experimental Cardiology Laboratory, University Medical Center Utrecht, 3584 CX Utrecht, The Netherlands; jsluijter@umcutrecht.nl; 4 Medical Physiology, University Medical Center Utrecht, 3584 CX Utrecht, The Netherlands; a.a.b.vanveen@umcutrecht.nl; 5 Tytgat Institute, Academic Medical Center, 1105 BK Amsterdam, The Netherlands; j.seppen@amc.uva.nl; 6 Department of Medical Biology, Amsterdam University Medical Centers, Academic Medical Center, 1105 AZ Amsterdam, The Netherlands; v.m.christoffels@amc.uva.nl

**Keywords:** pacemakers, gene therapy, cell therapy, progenitor cells, HCN channels

## Abstract

Sustained pacemaker function is a challenge in biological pacemaker engineering. Human cardiomyocyte progenitor cells (CMPCs) have exhibited extended survival in the heart after transplantation. We studied whether lentivirally transduced CMPCs that express the pacemaker current *I_f_* (encoded by *HCN4*) can be used as functional gene delivery vehicle in biological pacing. Human CMPCs were isolated from fetal hearts using magnetic beads coated with Sca-1 antibody, cultured in nondifferentiating conditions, and transduced with a green fluorescent protein (*GFP*)- or HCN4-GFP-expressing lentivirus. A patch-clamp analysis showed a large hyperpolarization-activated, time-dependent inward current (−20 pA/pF at −140 mV, n = 14) with properties typical of *I_f_* in HCN4-GFP-expressing CMPCs. Gap-junctional coupling between CMPCs and neonatal rat ventricular myocytes (NRVMs) was demonstrated by efficient dye transfer and changes in spontaneous beating activity. In organ explant cultures, the number of preparations showing spontaneous beating activity increased from 6.3% in CMPC/GFP-injected preparations to 68.2% in CMPC/HCN4-GFP-injected preparations (*P* < 0.05). Furthermore, in CMPC/HCN4-GFP-injected preparations, isoproterenol induced a significant reduction in cycle lengths from 648 ± 169 to 392 ± 71 ms (*P* < 0.05). In sum, CMPCs expressing HCN4-GFP functionally couple to NRVMs and induce physiologically controlled pacemaker activity and may therefore provide an attractive delivery platform for sustained pacemaker function.

## 1. Introduction

Biological pacing based on gene and cell therapy technologies has been the subject of intensive research, focused primarily on treating bradycardia. Several gene-based strategies are particularly promising; however, their potential clinical application depends on a demonstration of efficacy, safety, and durability of effect [1,2].

A major bottleneck in developing gene-based delivery platforms is long-term pacemaker function. Adenoviral vectors have been highly valuable for proof-of-principle studies; however, the effect persists only over the course of several weeks as successfully transduced cells are efficiently cleared by the immune system [3,4,5]. Adeno-associated virus (AAV) vectors have been extensively studied for myocardial gene therapy, can sustain prolonged in vivo transgene expression, and are currently used in clinical trials that target heart failure [6]. A disadvantage of AAV is their limited transgene capacity of ~3.5 kilobases (kb; excluding the promoter and regulatory elements), which precludes the use of larger genes, such as the skeletal muscle sodium channel SkM1 (5.5 kb) [7]. For larger genes, induced cell fusion could provide a nonviral alternative, although this technique currently relies on the use of polyethylene glycol [8], which is toxic and can induce an uncontrollable fusion of multiple cell and intracellular membranes [9]. Lentivirus (LV) vectors have a much larger transgene capacity (~8.0 kb) and can also generate long-term in vivo cardiac gene expression [10,11]. In contrast to AAV, LV vectors integrate into the target cell genome, resulting in long-term gene expression. The safety concerns surrounding LV, together with a relatively laborious production process, have delayed the use of LV vectors for in vivo gene transfer [12]. Recently, however, these issues have been largely addressed by the design of vectors with an LV genome that is largely devoid of virulence factors and divided over several plasmids for production [13], and by careful monitoring of preclinical animal models and human patients after LV gene therapy. One of these concerns was the risk of unintentional recombination, potentially leading to a replication-competent LV vector (RCL). No relevant RCL quantities were found in animals subjected to cell therapy with LV-transduced stem cells [14,15], or in long-term clinical studies on patients undergoing gene therapy with LV [16,17]. Another concern was the potential for insertional mutagenesis that could lead to neoplasia, especially in the hematopoietic system. Also, this can be re-evaluated now that long-term preclinical studies on model animals [14,18] and clinical trials [16,19] have not identified an increased cancer risk. For ex vivo modification of hematopoietic stem cells, LV vectors are a powerful tool, and have successfully been used in various clinical trials targeting blood and immune disorders [20]. At this stage, LV vectors therefore represent a logical choice for the ex vivo modification of biological pacemaker stem cells.

Genetically modified mesenchymal stem cells (MSCs) provide efficient delivery of a hyperpolarization-activated cyclic nucleotide-gated ion channels (HCN)-mediated pacemaker function to adjacent cardiomyocytes [21,22]. When MSCs are used as a delivery platform for pacemaker currents, they are typically undifferentiated and form gap junctions with myocardial cells, thereby providing an ion channel function to recipient tissue [22]. HCN2-expressing MSCs that were transplanted into left ventricular epicardium of dogs with atrioventricular block generated an autonomically controlled pacemaker function that persisted throughout a 6-week study [23]. However, longer studies have been problematic, most probably because the MSCs were lost from the injected area over time [1].

We therefore developed a cell delivery platform with a better potential for long-term intra-myocardial persistence. We selected human cardiomyocyte progenitor cells (CMPCs), since they are native to the human heart and can persist for months after intra-myocardial transplantation [24]. In the present study, we studied lentiviral transduction of CMPCs, and evaluated their functional interaction with cardiomyocytes, as well as the ability of HCN4-expressing CMPCs to generate an autonomically controlled pacemaker function.

## 2. Results

### 2.1. CMPCs are Efficiently Transduced by LV Vectors

**Figure 1:**
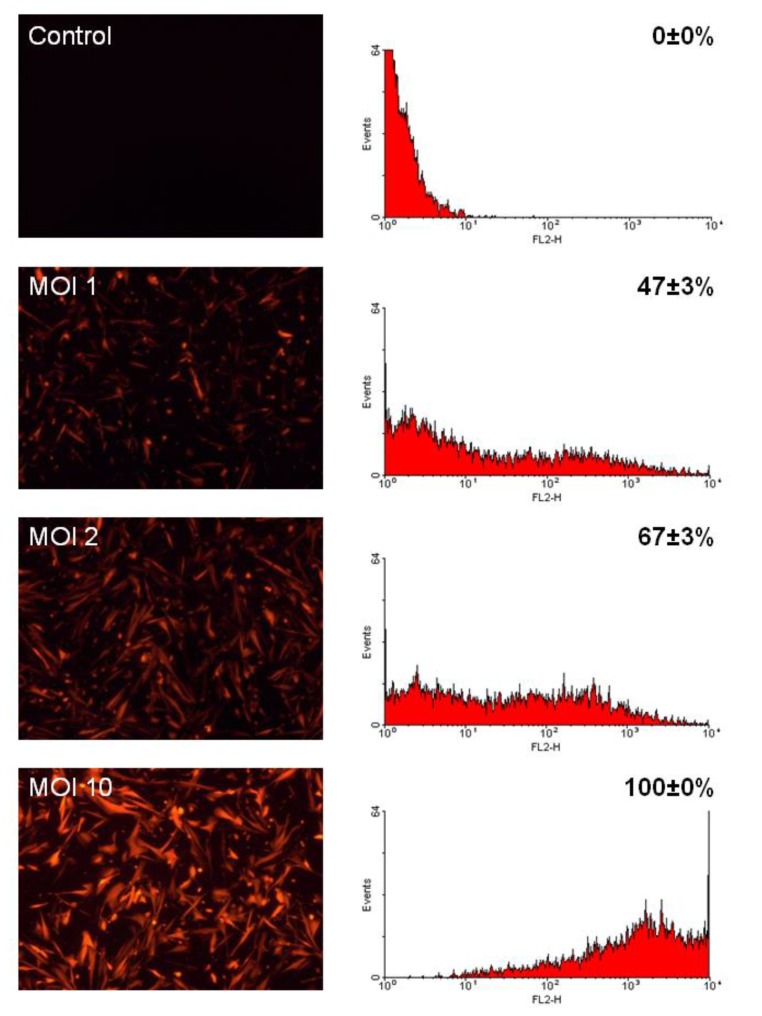
Efficient transduction of cardiomyocyte progenitor cells (CMPCs) with lentivirus (LV) vectors. CMPCs were transduced with LV-DsRed at multiplicities of infection (MOIs) of 1, 2, and 10. Four days after transduction, cells were assessed by fluorescence microscopy (left) and flow cytometry (right). Histogram plots demonstrate that the proportion of DsRed-positive cells increases as a function of the MOI. Transduction efficiency is presented as mean ± standard error of the mean (SEM) (n = 3).

To determine the ability of LV vectors to provide gene transfer to CMPCs, we transduced the cells with LV expressing the red fluorescent protein DsRed at varying multiplicities of infection (MOI). Efficiency of gene transfer was assessed by fluorescence microscopy and flow cytometry (Figure 1). CMPCs were easily transduced. MOIs of 1, 2, and 10 resulted in respectively 47%, 67%, and 100% DsRed expression of the cells. The mean DsRed fluorescence intensity increased 10-fold when the MOI was increased from 2 to 10.

When we transduced CMPCs with LV expressing HCN4 and GFP, we observed toxicity at MOIs of 10 and higher. Toxicity was identified by a reduced cytoplasmic volume in combination with poor cell survival and growth of HCN4-expressing cells. Since this degree of gene toxicity did not occur when CMPCs were transduced with LV-HCN4-GFP at an MOI of 2, we used this MOI in follow-up experiments, resulting in 65–70% transduction.

### 2.2. Undifferentiated CMPCs Couple to Cardiomyocytes

**Figure 2:**
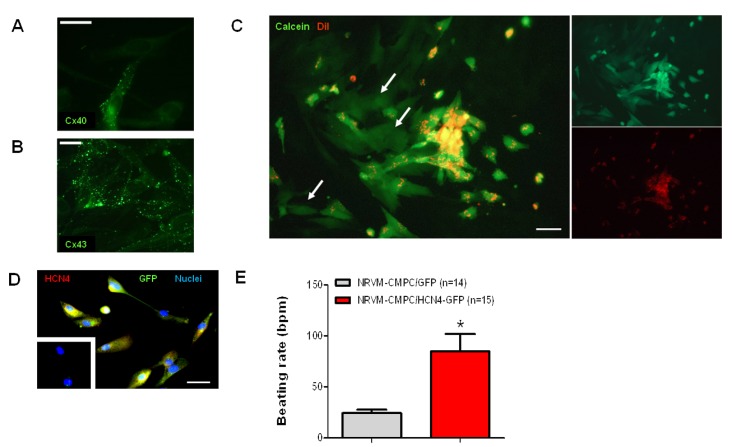
Functional coupling of CMPCs to cardiomyocytes. (**A**,**B**) Immunolabeling of connexin proteins Cx40 and Cx43. Scale bars represent 30 and 25 µm, respectively. (**C**) Fluorescent microscopy of dye transfer from CMPCs labeled with DiI (red) and Calcein (green) to unlabeled cardiomyocytes. Cardiomyocytes that have imported Calcein are indicated by white arrows. Scale bar represents 45 µm. (**D**) Immunolabeling of GFP (green) and HCN4 (red) in CMPCs transduced with LV-HCN4-GFP. Yellow indicates co-staining. Nuclei were counterstained blue with DAPI. Non-transduced CMPCs, shown in the inset, stained negative for GFP and HCN4. Scale bar represents 45 µm. (**E**) Average beating rates of neonatal rat ventricular myocyte (NRVM) monolayers co-cultured with CMPCs expressing GFP alone (grey) and HCN4 and GFP (red). * indicates *P* < 0.05.

Gap junctional coupling between CMPCs and cardiomyocytes is essential for cardiac delivery of ion channel function. We therefore studied the expression of the two major cardiac gap junction proteins Connexin (Cx)40 and Cx43 in CMPCs. Using immunofluoresence, we found that Cx43 is the predominant gap junctional protein in CMPCs, whereas Cx40 is expressed at lower levels (Figure 2A,B). Next, the functionality of CMPC-to-cardiomyocyte coupling via gap junctions was assessed by dye transfer. CMPCs were loaded with Calcein-AM and permanently stained with the membrane dye DiI. The Calcein-AM ester is hydrolyzed in the cytoplasm by nonspecific esterases to produce polyanionic Calcein. Calcein trapped inside the CMPC can only be transferred to cardiomyocytes via gap junctions. Figure 2C shows the spread of the Calcein dye from DiI-stained CMPCs to adjacent cardiomyocytes, proving that functional gap junctions were formed between both cell types.

Functional cell interaction was further studied using HCN4-expressing CMPCs. HCN4 expression was first demonstrated via immunofluoresence (Figure 2D). Untransduced CMPCs were negative for HCN4 staining (Figure 2D; inset). CMPCs transduced with LV-HCN4-GFP or LV-GFP were seeded on top of neonatal rat ventricular myocyte (NRVM) monolayers. After seven days of culturing, spontaneous beating rates were significantly faster in cardiomyocyte-CMPC/HCN4-GFP co-cultures than in cardiomyocyte-CMPC/GFP co-cultures (*P* < 0.05; Figure 2E).

### 2.3. I_f_ Properties in LV-Transduced CMPCs

**Figure 3:**
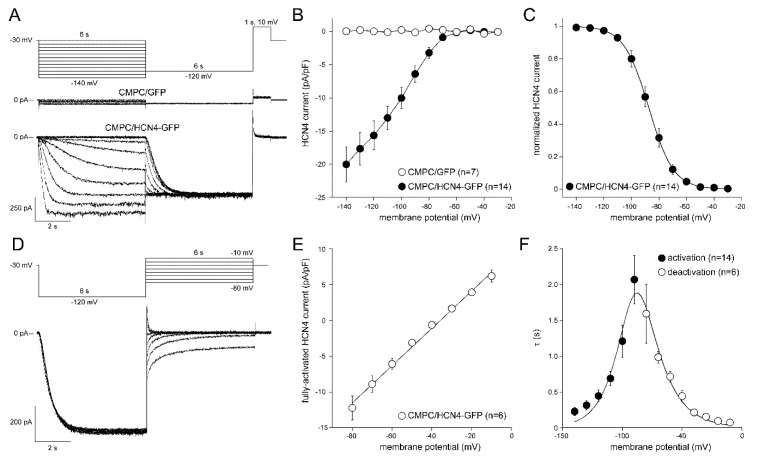
Pacemaker current, *I_f_*, in single LV-transduced CMPCs. (**A**) Voltage steps and typical *I_f_* traces of CMPC/GFP and CMPC/HCN4-GFP cells. Inset: voltage pulse protocol to measure activation properties. (**B**) Average current-voltage (I–V) relationships of *I_f_* of CMPC/GFP and CMPC/HCN4-GFP cells. (**C**) Voltage dependence of *I_f_* activation. Solid line is the Boltzmann fit to the experimental data. (**D**) Typical *I_f_* traces of CMPC/HCN4-GFP cells. Inset: voltage pulse protocol to measure deactivation properties. (**E**) I–V relationship of the fully-activated HCN4 current. Solid line is the linear fit to the experimental data. (**F**) Time constants of (de)activation. Solid line is the best fit curve to the equation τ = 1/[A1 × exp(−V/B1) + A2 × exp(V/B2)], where τ is the activation or deactivation kinetic time constant, and A1, A2, B1, and B2 are calculated fitting parameters [25].

To study the properties of the *HCN4*-encoded pacemaker current *I_f_*, CMPCs were transduced with either LV-GFP or LV-HCN4-GFP at an MOI of 2, and cultured for 7 to 14 days. Figure 3A shows typical *I_f_* recordings from single CMPCs. Average current-voltage (I–V) relationships of *I_f_* are summarized in Figure 3B. In CMPCs expressing GFP alone, we observed only small amplitude leak currents during the 6-s-long hyperpolarizing steps (Figure 3A); time-dependent components were not observed (Figure 3B). CMPCs expressing HCN4-GFP showed large time-dependent inward currents in response to the hyperpolarizing voltage steps, typical for *I_f_* (Figure 3A,B). To analyze voltage-dependence of activation of CMPC/HCN4-GFP cells in detail, we plotted the normalized tail current amplitude against the preceding hyperpolarizing potential and fitted a Boltzmann function to the data. Average curves are shown in Figure 3C. The average V_1/2_ and k of the Boltzmann fit to the data were −86.7 ± 2.1 and −7.7 ± 0.8 mV, respectively. The voltage-dependence of the fully activated current was evaluated over a large range of potentials (−80 to −10 mV) by measuring the tail current amplitudes after a hyperpolarizing pulse to −120 mV. Figure 3D shows a typical example, and Figure 3E shows the average I–V relationship of fully activated HCN4. The average reversal potential was −35.7 ± 0.8 mV (n = 6). Activation and deactivation time constants (Figure 3F) were obtained from mono-exponential fits of the step (Figure 3A) and tail (Figure 3D) currents, respectively.

**Figure 4:**
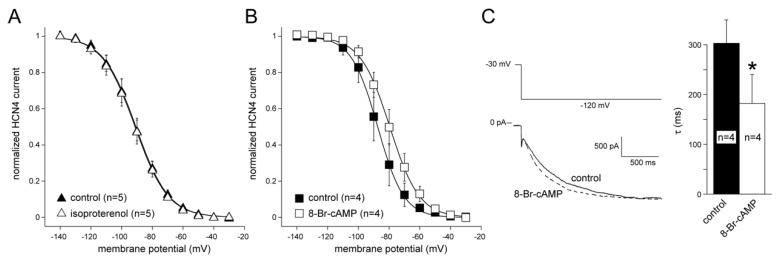
*I_f_* modulation in CMPCs expressing HCN4-GFP. (**A**) Effects of 10 nM Isoproterenol on voltage dependency of *I_f_* activation. (**B**) Effects of 1 mM 8-Br-cAMP on voltage dependency of *I_f_* activation and speed of *I_f_* activation at −120 mV. (**C**) typical *I_f_* traces at –120 mV in the absence and presence of 8-Br-cAMP. * indicates *P* < 0.05.

In a final series of patch-clamp experiments, we tested the effects of adrenoreceptor and cAMP stimulation on basic *I_f_* properties in CMPC/HCN4-GFP cells. The voltage-dependence of activation was measured before and 5 min after application of 10 nM of the epinephrine analogue isoproterenol or 1 mM 8-Br-cAMP (a cAMP analogue). Neither *I_f_* voltage dependency (Figure 4A) nor speed of *I_f_* activation (data not shown) were affected by isoproterenol. However, 8-Br-cAMP did cause a positive shift in the voltage dependence of *I_f_* activation (Figure 4B). On average, V_1/2_ of activation was 8.5 ± 2.2 mV (n = 4; *P* < 0.05) more positive compared to the control condition. Additionally, 8-Br-cAMP accelerated the current activation significantly. Figure 4C, left panel, shows a typical example of *I_f_* activated upon a hyperpolarizing step to −120 mV in the absence and presence of 8-Br-cAMP. On average, the activation time (upon steps to −120 mV) decreased by 40 ± 16% in response to cAMP (Figure 4C, right panel).

### 2.4. Biological Pacemaker Function in Organ Explant Cultures

**Figure 5:**
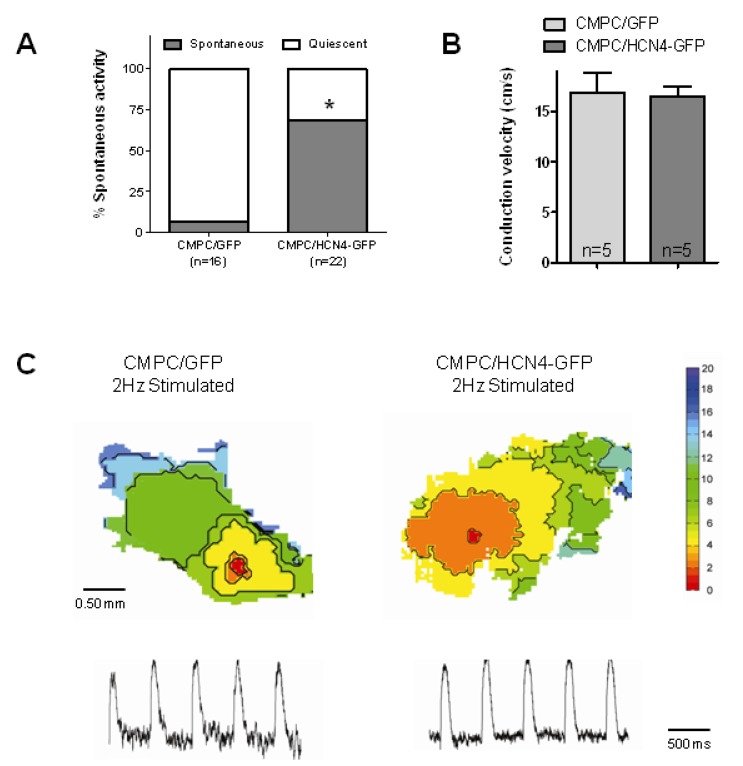
Baseline properties of organ explant cultures injected with CMPCs. (**A**,**B**) Summary data of spontaneous beating activity and conduction velocity of explants injected with CMPC/GFP and CMPC/HCN4-GFP. (**C**) Typical activation maps and optical action potentials measured during stimulation at 2 Hz. * indicates *P* < 0.05.

To further study the pacemaker properties of CMPCs expressing HCN4-GFP, we injected CMPC/HCN4-GFP and control CMPC/GFP into organ explant cultures. Injection of the CMPCs in this neonatal rat cardiac tissue resulted in a significant increase in spontaneous beating activity in explants with CMPC/HCN4-GFP (*P* < 0.05; Figure 5A). We next tested whether the speed of impulse propagation was affected by HCN4 expression. We found that conduction velocities during electrical stimulation were comparable in explants injected with CMPC/HCN4-GFP or CMPC/GFP cells (Figure 5B,C). These conduction velocities also did not differ significantly from previously reported unmodified organ explant cultures [26].

**Figure 6:**
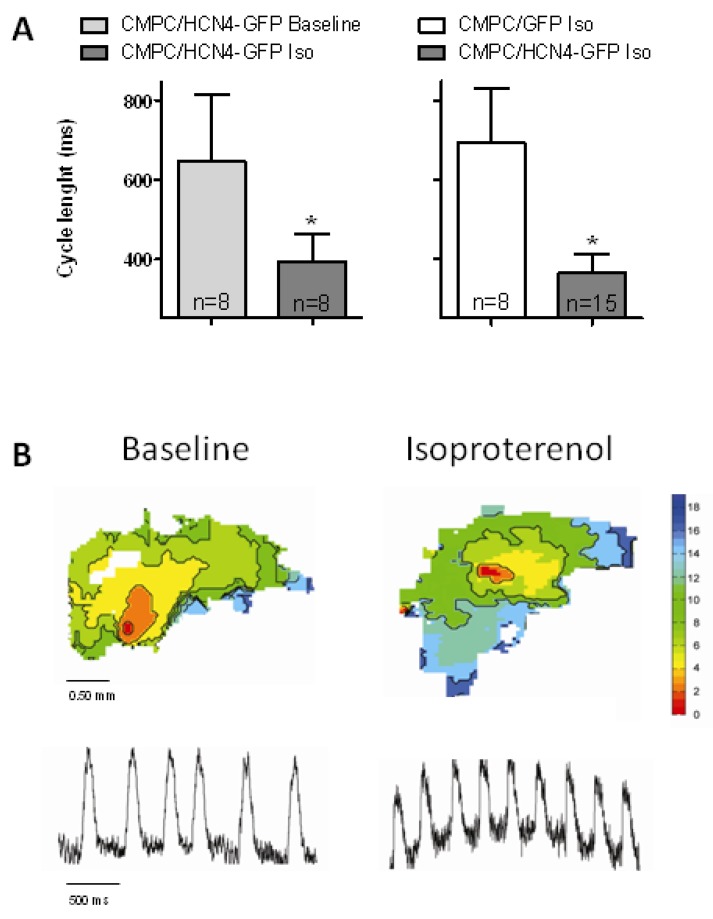
Autonomic modulation of pacemaker function in explants injected with CMPC/HCN4-GFP cells. (**A**) Isoproterenol (10 nM) significantly shortens cycle lengths in explants injected with CMPC/HCN4-GFP (left panel). In the presence of isoproterenol, cycle lengths are significantly shorter in explants injected with CMPC/HCN4-GFP than in CMPC/GFP-injected explants (right panel). (**B**) Typical activation maps and optical action potentials in CMPC/HCN4-GFP-injected explants at baseline and during superfusion with isoproterenol (two different preparations). * indicates *P* < 0.05.

Finally, to test the response to autonomic modulation in explants injected with CMPC/HCN4-GFP cells, we exposed them to 10 nM isoproterenol and found that all explants showed spontaneous beating activity. Moreover, cycle lengths shortened (*P* < 0.05 versus baseline) and were significantly faster in CMPC/HCN4-GFP than in CMPC/GFP-injected explants (*P* < 0.05; Figure 6). In explants injected with CMPC/GFP cells, isoproterenol increased the fraction of spontaneously beating preparations to only 76% (*P* not significant versus CMPC/HCN4-GFP). In comparison, previous research has shown spontaneous activity in 28% of non-injected organ explants, with cycle lengths of 468 ± 97 ms [26]. Addition of isoproterenol was not tested in these non-injected organ explants.

## 3. Discussion

This study investigated biological pacemakers constructed from LV-transduced CMPCs. An in vitro analysis indicated that CMPCs were efficiently transduced by LV vectors and expressed connexins, leading to increased spontaneous activity in cardiac myocytes coupled to HCN4-overexpressing CMPCs. Injection of CMPC/HCN4-GFP into organ explant cultures caused pacemaker activity with intact autonomic modulation. Because LV vectors integrate into the host genome, and CMPCs have been shown to persist after transplantation into myocardium, this approach provides a new and promising strategy for long-term biological pacing.

### 3.1. CMPC Transduction

In a CMPC-based biological pacemaker system, only HCN4-expressing cells contribute to pacemaker function. We therefore sought the highest achievable ratio of these cells. Although a transduction efficiency of 100% was readily achieved with LV-DsRed (MOI of 10; Figure 1), this protocol could not be applied to LV-HCN4-GFP due to dose-dependent gene toxicity. However, at an MOI of 2, 65–70% of the cells were transduced. This is well above the 30–45% obtained after electroporating MSCs with plasmid DNA carrying HCN2 [22]. Given the level of in vivo function generated by these gene-modified MSCs [23], we expect that our LV transduction protocol does not necessarily require further optimization. Yet, improved outcomes may potentially be obtained by sorting for successfully gene-modified CMCPs.

### 3.2. Functional Interaction Between CMPCs and Cardiomyocytes

After one week, GFP-positive CMPCs were found in the organ explants (Supplemental Figure S1). To demonstrate functional interaction between CMPCs and cardiomyocytes, we performed a dye transfer experiment (Figure 2C) and studied the beating rate of NRVM monolayers co-cultured with CMPCs expressing either HCN4-GFP or GFP (Figure 2E). Although these experiments showed functional interaction between CMPCs and cardiomyocytes, they did not provide a true quantification of gap junction coupling between both cell types. Previous experiments by Valiunas and coworkers showed a sigmoid curve when plotting the transmitted HCN2 current and the junction conductance between cardiomyocytes and MSCs [27], indicating that high gap junction conductance is needed for high HCN current delivery. Previous research has found high Cx43 levels expressed by CMPCs, providing functional coupling with cardiomyocytes [28,29,30]. Accordingly, given the connexin expression levels (Figure 2A,B), the pronounced dye transfer (Figure 2C), and the significant increases in spontaneous beating activity (Figure 2E and Figure 5A), it seems likely that the gap junction conductance between CMPCs and cardiomyocytes was adequate.

Previous work has shown that NRVMs and MSCs can fuse within several hours after co-culture [31]. Although we did not investigate the occurrence of cell fusion in detail, the outcomes of our dye-transfer experiment are better compatible with metabolic coupling between CMPCs and NRVMs, than with cell fusion.

### 3.3. HCN4 Expression and Modulation

Patch-clamp experiments were performed to evaluate the properties of HCN4 protein expressed in CMPCs. These experiments confirmed functional HCN4 expression, with typical *I_f_* activation and inactivation kinetics. The V_1/2_ of HCN4 activation was more negative in CMPCs than the kinetics of HCN4 expressed in NRVMs that were previously reported [32], and this may have contributed to a lower peak current density in CMPCs. However, these differences are small and comparable to differences found between HCN2 expressed in MSCs and in NRVMs [22,33]. Despite these differences, in vivo function based on HCN2 function delivered by virus or by stem cells was similar [23,34], suggesting similar outcomes with CMPCs.

A difference between MSCs and CMPCs is the direct sensitivity to beta-adrenergic signaling in MSCs. While MSCs expressing HCN2 showed a positive shift of voltage-dependent activation upon beta-adrenergic stimulation [22], such effects were absent in CMPCs expressing HCN4 (Figure 4A), despite their sensitivity to cAMP (Figure 4B). Theoretically, this could be a downside of CMPCs in the context of biological pacemaker engineering. However, as CMPCs and cardiomyocytes function together as a combined pacemaker unit, beta-adrenergic stimulation can still activate the downstream cascade within the cardiomyocyte component. Here, higher cAMP levels regulate a variety of ion channels and Ca^2+^-handling proteins that support a faster rate [35,36,37]. Furthermore, cAMP passes through gap junctions [38] and may thus enhance HCN4 channel activity in CMPCs. Such mechanisms likely explain the significant autonomic modulation found in the CMPC/HCN4-GFP-injected organ explant cultures (Figure 6).

### 3.4. Biological Pacemaker Function Induced by HCN4-Expressing CMPCs

The level of baseline function generated by CMPC/HCN4-GFP cells injected into organ explant cultures was comparable to what we previously reported for organ explants co-cultured with spontaneously active NRVMs [26]. As suggested above, we expect a similar level of in vivo function as previously reported for MSC-based delivery of HCN2 or HCN4 function [23,39,40]. These in vivo MSC studies showed suboptimal basal and maximal beating rates, causing significant dependence on electronic back-up pacing [23,39,40], and loss of pacemaker function over time [1]. However, we have recently shown that HCN-based biological pacemaker function is significantly enhanced by co-expression of the skeletal muscle sodium channel SkM1. In this approach, HCN channels generate slow diastolic depolarization and SkM1 hyperpolarizes the action potential threshold, thereby improving the rate and stability of pacemaker function [7]. Furthermore, in vitro and in vivo studies have demonstrated that SkM1 current can also be efficiently delivered via undifferentiated MSCs [41,42]. This suggests that CMPC/HCN4-based biological pacemaker function can be significantly improved with SkM1 co-delivery.

A final consideration is the longevity of biological pacing based on gene-modified CMPCs, since this depends on persistent gene expression and cell localization in the injected area. Other studies suggest that LV vectors are perfectly suitable for stable gene modification of stem cells, especially if specific promoters are used [20]. As for cell persistence in the injection site, we found substantial retention of our CMPCs for the duration of a 3-month study on myocardial infarction zone transplantation in mice [24]. Since biological pacemakers are typically applied to relatively healthy myocardium, we expect comparable or better cellular persistence after CMPC transplantation in this setting.

### 3.5. Safety Issues Related to CMPC-Based Biological Pacemakers

The potential risk for pro-arrhythmia is a relatively unaddressed issue of biological pacemakers [1]. Some pacemaker constructs generated spontaneous tachycardia [43,44]. In addition, the approaches that did not cause spontaneous arrhythmias still have to be tested in pathological settings, such as ischemia, myocardial infarction, or heart failure. Only after these studies, we can assess the risk-benefit ratio of biological pacing for the full range of strategies and delivery platforms, including the CMPC vehicle described here.

The use of allogenic fetal cells in this study makes the use of CMPCs as biological pacemakers more complicated. It is possible to isolate CMPCs from adult atrial appendages [28], overcoming ethical issues regarding the use of fetal cells. Moreover, such patient-specific cell isolation can provide for an autologous cell source, thereby bypassing potential immunological issues.

Another safety issue that needs consideration is the risk for insertional mutagenesis and tumor formation. This risk can efficiently be reduced by the use of third-generation self-inactivating LV vectors, as applied in this study. In these vectors, the U3 transcriptional control elements from the long terminal repeats are not present. This deletion removes potential oncogene activation by these elements and makes rescue by wild-type viruses impossible [45,46]. The potential risk for oncogene activation and tumor formation by long-range transcription from the LV transgene promoter is very low because of the low number of cells typically used (e.g., ~1 million) and the limited number of cell divisions after introduction of the therapeutic gene(s) [23]. This is different from the clinical trials in which a small number of LV-transduced cells were used to repopulate the whole bone marrow. Oncogene activation can be further reduced by the use of promoters with weak-to-moderate activity [47]. Still, before clinical testing of CMPC-based biological pacemakers, a thorough evaluation of potential insertional mutagenesis and tumor formation will be essential.

## 4. Materials and Methods

### 4.1. Cardiac Progenitor Cells Isolation and Culture

CMPCs were isolated from human fetal hearts obtained after elective abortion, as previously described [48]. After approval from the Institutional Review Board of the University Medical Center Utrecht (ethical code number DCA 100582), hearts were collected after individual informed consent. In brief, hearts were isolated and perfused using a Langendorff perfusion setup [49]. After digestion with collagenase and protease, CMPCs were isolated from the non-myocyte fraction of the cell suspension using magnetic cell sorting beads coupled to Sca-1 antibody (Miltenyi Biotech, Bisley, Surrey, United Kingdom, cat. no. 130-091-176). Cells were cultured in nondifferentiating conditions as described previously [48], namely on 0.1% gelatin-coated material, using SP++ medium (EBM-2 with EGM-2 additives (Lonza, Basel, Switzerland, cat. no. CC-3162), mixed 1:3 with M199 (Gibco, Grand Island, NY, USA, cat. no. 31150-030)) supplemented with 10% fetal calf serum (Gibco, cat. no. 10270-106), 10 ng/mL basic fibroblast growth factor, 5 ng/mL epithelial growth factor, 5 ng/mL insulin-like growth factor, and 5 ng/mL hepatocyte growth factor. CMPCs were cultured in these nondifferentiating conditions for a maximum of 20 passages.

### 4.2. LV Vectors, Transduction, and Efficiency

The bicistronic LV-HCN4 expression vector has previously been described [32]. Briefly, HCN4 expression is controlled by a cytomegalovirus (CMV) promoter and linked to green fluorescent protein (GFP) expression by an internal ribosome entry site from encephalomyocarditis virus. LV vectors with CMV-driven GFP and DsRed genes served as control vectors. The vectors are designated LV-HCN4-GFP, LV-GFP, and LV-DsRed, respectively. LV particles were generated by co-transfection of HEK293T cells, and concentrated and titrated as previously described [50].

CMPCs were transduced in the presence of 8 μg/mL polybrene (Sigma-Aldrich, St. Louis, MO, USA, cat.no. TR-1003), with LV-HCN4-GFP or LV-GFP at an MOI of 2. Transduced cells were used after 4 days for co-culture and organ explant experiments, or within 7–14 days for patch-clamp analysis. To assess the LV transduction efficiency, 5 × 10^4^ CMPCs were transduced as above with LV-DsRed at MOIs of 1, 2, and 10. The cells were washed twice in phosphate-buffered saline (PBS), trypsinized, resuspended in SP++ medium, and analyzed using a Becton Dickinson FACSCalibur (BD Biosciences, Franklin Lakes, NJ, USA).

### 4.3. Cell Isolation and Co-Culture of Neonatal Rat Ventricular Cardiac Myocytes

All animal experiments were performed in accordance with the Guide for the Care and Use of Laboratory Animals published by the National Institute of Health (NIH Publication No. 85-23, revised 1996), and approved by the Academic Medical Center committee for animal experiments. Six to twelve neonatal rats were sacrificed per procedure, as described previously [32,51]. NRVMs were cultured in M199 (Gibco, cat.no. 31150-030) containing (mM): 137 NaCl, 5.4 KCl, 1.3 CaCl_2_, 0.8 MgSO_4_, 4.2 NaHCO_3_, 0.5 KH_2_PO_4_, 0.3 Na_2_HPO_4_, and supplemented with 20 units/100 mL penicillin, 20 μg/100 mL streptomycin, 2 μg/100 mL vitamin B12, and 5% neonatal calf serum, except on the first day or culture, when medium containing 10% neonatal calf serum was used. The cells were cultured on collagen-coated glass coverslips at 37 °C in 1% CO_2_.

### 4.4. Immunofluoresent Labeling

For the connexin staining, untransduced cells were fixed in methanol (−20 °C) or 4% paraformaldehyde (Sigma-Aldrich, cat.no. P6148), washed with PBS (homemade), and permeabilized with 0.2% Triton X-100 (Merck, Darmstadt, Germany, cat. no. 108603) in PBS. Nonspecific binding of antibodies was blocked with 2% bovine serum albumin (Sigma-Aldrich, cat.no. 05479). Incubation with primary antibodies was performed overnight in PBS with 10% normal goat serum. The antibodies used recognized Cx40 (Rb polyclonal, Chemicon, Burlington, MA, USA, cat. no. AB1726) or Cx43 (Rb polyclonal, Zymed, Wien, Austria, cat. no. 71-0700). Immunolabeling was performed using FITC-conjugated secondary antibodies (Jackson Laboratories, Ely, United Kingdom, Donkey-α-goat, cat. no. 211-095-109 and Goat-α-Rb, cat. no. 705-095-147). All incubation steps were performed at room temperature and, in between all incubation steps, cells were washed with PBS. Finally, cells were embedded with Vectashield mouting medium with DAPI (Vector Laboratories, Peterborough, United Kingdom, cat. no. H-1200).

For the HCN4 staining, cells were fixed 4 days after transduction with methanol:acetone (4:1) and washed with 0.05% Tween-20 in PBS. As a negative control, non-transduced cells were prepared in parallel. For HCN4 detection, anti-HCN4 goat polyclonal IgG (Santa Cruz Biotechnology, Heidelberg, Germany, cat.no. SC-19714) was used as primary, and donkey anti-goat IgG conjugated with Alexa 594 (Invitrogen, Waltham, MA, USA, cat. no. A11058) as secondary antibody. For GFP detection, anti-GFP rabbit polyclonal IgG (Invitrogen, cat.no. A-11122) and goat anti-rabbit IgG conjugated with Alexa 488 (Invitrogen, cat.no. A-11034) were used, respectively. The cells were subsequently embedded in Vectashield with DAPI (Vector Laboratories, Peterborough, United Kingdom, cat. no. H-1200).

### 4.5. CMPC-to-Cardiomyocyte Dye Transfer

CMPC-to-cardiomyocyte coupling via gap junctions was assessed by dye transfer of Calcein between CMPCs and cardiomyocytes. CMPCs were loaded with 5 μM DiI (Invitrogen, cat. no. D282) and 5 μM Calcein-AM (Invitrogen, cat. no. C3099). CMPCs were then trypsinized and added to a NRVM monolayer. Imaging was performed after 6 h of co-culture using an inverted fluorescence microscope (Leica DMIL, Leica Microsystems, Wetzlar, Germany) with a DFC320 camera (Leica Microsystems, Wetzlar, Germany). Calcein was imaged with 450 to 490 nm fluorescence excitation and 500 to 550 nm emission. DiI was imaged with 515 to 560 nm excitation and >590 nm emission.

### 4.6. Functional Interaction Between CMPCs and Cardiomyocytes

CMPCs transduced with LV-HCN4-GFP or LV-GFP were seeded on top of 6-day-old NRVM monolayers. Seven days after the initiation of co-culture, spontaneous beating rates were assessed by counting the contractions during 1 min. These cultures were superfused with Tyrode’s solution (36 ± 0.2 °C) containing (mM): NaCl 140, KCl 5.4, CaCl_2_ 1.8, MgCl_2_ 1.0, glucose 5.5, HEPES 5.0; pH 7.4 (NaOH).

### 4.7. Single Cell Measurements

For patch-clamp experiments, single CMPCs were harvested from the culture flask by trypsinization, stored in SP++ medium at room temperature (20 °C), and studied within 3 h. Cell suspensions were put into a recording chamber on the stage of an inverted microscope (Nikon Diaphot, Melville, New York, NY, USA) and superfused with Tyrode’s solution (36 ± 0.2 °C). Single CMPCs that visibly formed branches with the bottom of the recording chamber and exhibited green fluorescence were selected for electrophysiological measurements.

*I_f_* was recorded using the amphotericin-perforated patch-clamp technique and an Axopatch 200B amplifier (Molecular Devices Corporation, Sunnyvale, CA, USA). Signals were low-pass filtered (cut-off frequency 5 kHz) and digitized at 5 kHz. Series resistance was compensated by ≥80%, and potentials were corrected for the estimated 15 mV change in liquid-junction potential. Voltage control, data acquisition, and analysis were accomplished using custom-made software. Laboratory-made pipettes (2–3 MΩ resistance; borosilicate glass) were filled with solution containing (mM): K-gluc 125, KCl 20, NaCl 10, amphotericin-B 0.22, HEPES 10; pH 7.2 (KOH). Cell membrane capacitance was determined as described previously [52]. *I_f_* was measured during 6-s hyperpolarizing steps (range −30 to −140 mV) from a holding potential of −30 mV. Next, a 6-s step to −120 mV was applied to record tail current followed by a 1-s pulse to 10 mV to ensure full deactivation (see Figure 3A for protocol; cycle length 18-s). Activation kinetics were measured during the 6-s hyperpolarizing steps. The current-voltage (I–V) relation was constructed from the current measured at the end of the 6-s hyperpolarizing steps. Currents were normalized to cell capacitance. Tail current, plotted against test voltage, provided the activation-voltage relation. The activation-voltage relation was normalized by maximum conductance and fitted with the Boltzmann function I/Imax = A/{1.0 + exp[(V_1/2_ – V)/k]} to determine the half-maximum activation voltage (V_1/2_) and slope factor (*k*). Deactivation kinetics and reversal potential (Erev) were measured during depolarizing steps (range −80 to −10 mV, duration 6 s) after a 6-s prepulse to −120 mV to ensure full activation (see Figure 3D for protocol; cycle length 15 s). The time course of *I_f_* (de)activation was fitted by the mono-exponential equation I/Imax = A × [1 – exp(–t/τ)], ignoring the variable initial delay in *I_f_* (de)activation [17,18]. The effects of 10 nM isoproterenol (Sigma-Aldrich) and 1 mM 8-Bromoadenosine 3′,5′-cyclic monophosphate (8-Br-cAMP; Sigma-Aldrich) on *I_f_* were measured at least 5 min after application of the drugs.

### 4.8. Organ Explant Cultures

We previously developed organ explant cultures as a screening tool for myocardial gene and cell therapies. An advantage of this system is the low-to-absent intrinsic automaticity, which makes it particularly useful to test novel approaches to biological pacing [32]. To obtain these cultures, 2-day-old Wistar rats (Charles River) were decapitated, and, after excision of the hearts, right ventricles were removed and used for further experiments. After isolation of tissue, 5 × 10^5^ CMPCs, transduced with either LV-HCN4-GFP or LV-GFP, were injected into the right ventricle. This tissue was placed, endocardial side down, on drained collagen gels and cultured. Organ explant cultures were left untreated overnight, after which M199 medium supplemented with 1% fetal bovine serum, 1% penicillin/streptomycin 100, 5 μg/mL insulin, 5 μg/mL transferrin, 5 ng/mL selenium, and 2 mM l-glutamine was added.

### 4.9. Optical Mapping of Impulse Generation and Action Potential Propagation

To assess spontaneous beating activity, we performed optical mapping experiments of organ explant cultures injected with CMPCs. Cultures were stained with 15 μM di- 4-ANEPPS (Invitrogen, cat. no. D1199) in EBSS with Ca^2+^ and Mg^2+^ (Gibco, cat. no. 24010043) for 7.5 min. After staining, explants were placed in an optical mapping apparatus in Tyrode’s solution (37.0 ± 0.5 °C). Excitation light was delivered by six power light-emitting diodes (LEDs, band-pass filtered 510 ± 20 nm). Emission fluorescence (filtered >610 nm) was transmitted through a tandem lens system on a Complementary Metal Oxide Semiconductor chip (Micam Ultima, SciMedia, Costa Mesa, CA, USA) with a spatial resolution of 100 × 100 pixels. Data acquisition was carried out with a sample frequency of 1 kHz. Subsequently, data were analyzed offline using custom-made software based on Matlab (Mathworks, Natick, MA, USA) [53]. We characterized explants by the presence of spontaneous beating activity, cycle length (CL) of spontaneous activity, and response to 10 nM isoproterenol (Sigma-Aldrich). In preparations with irregular spontaneous activity, we determined the average cycle length over five recordings of 5 s each.

### 4.10. Statistics

Data are expressed as mean ± standard error of the mean (SEM). Group comparisons were made using a Student’s *t*-test. Categorical data were compared using Fisher’s Exact test. The level of significance was set at *P* < 0.05.

## 5. Conclusions

CMPCs provide a novel ion channel current delivery platform, potentially of particular value for long-term modification of the cardiac substrate. As such, CMPCs provide an attractive alternative to direct LV gene transfer. CMPCs are therefore proposed as a powerful tool to treat bradycardias and other cardiovascular disease.

## Supplementary Materials

The following are available online. Figure S1: Presence of CMPCs in the organ explants.
