# Supplementary figures and images for: Cardiomyocyte Progenitor Cells as a Functional Gene Delivery Vehicle for Long-Term Biological Pacing
Source: Molecules. 2019 Jan 5;24(1):181. doi: 10.3390/molecules24010181 (PMC6337610; doi:10.3390/molecules24010181)

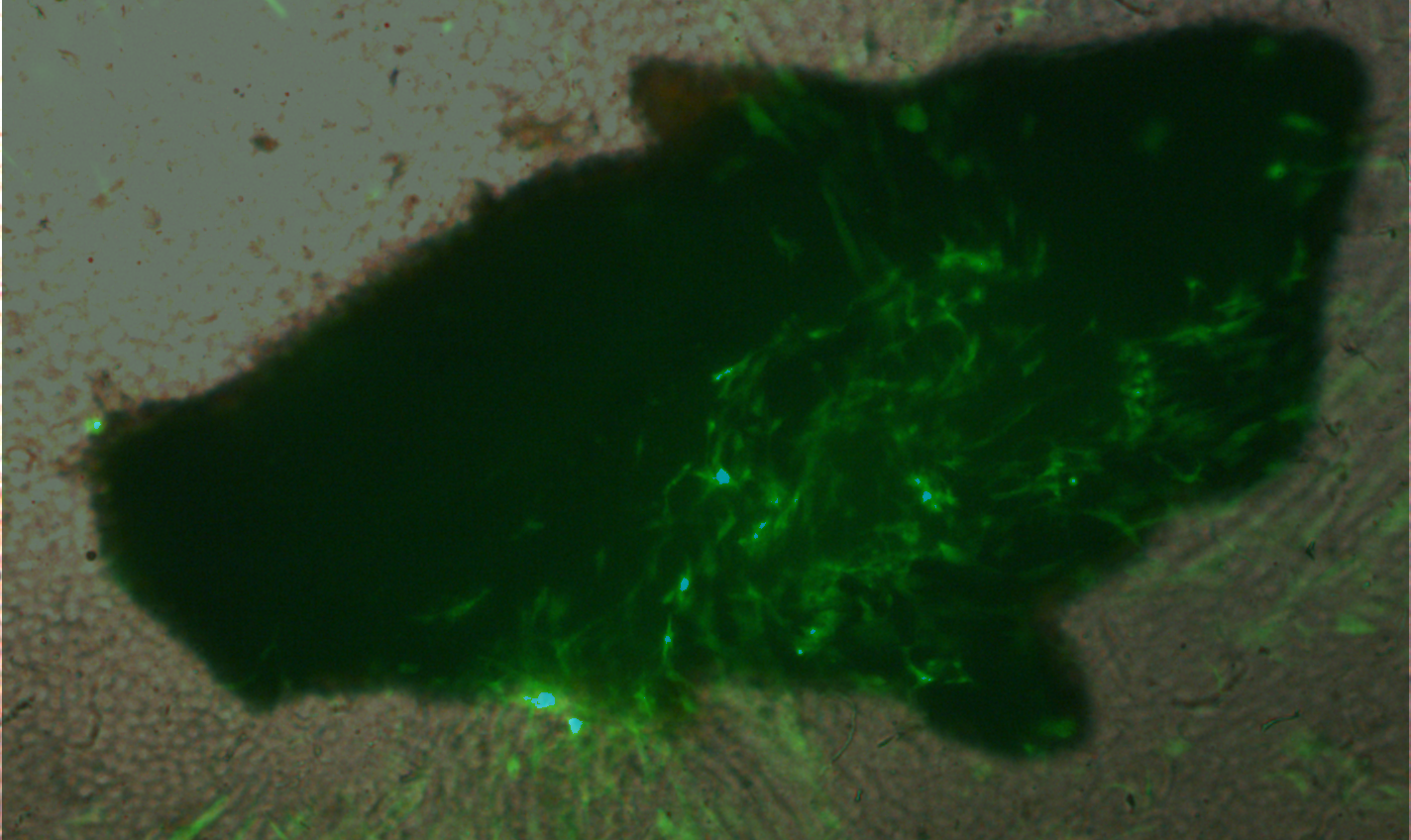

Supplement: Supplementary file 1 [file molecules-24-00181-s001.zip › molecules-406856-supplementary.png]
